# Acesulfame Potassium: Soffritti Responds

**Published:** 2006-09

**Authors:** Morando Soffritti

**Affiliations:** Cesare Maltoni Cancer Research Center, European Foundation of Oncology and Environmental Sciences, “B. Ramazzini”, Bologna, Italy, E-mail: crcfr@ramazzini.it

Karstadt makes an important point regarding the need for more adequate long-term carcinogenicity testing of the artificial sweetener acesulfame K. The issues raised in her letter stimulated me to offer some additional considerations.

As reported in a previous paper (Soffritti et al. 1999), one of the most important issues in environmental and industrial carcinogenesis is how to deal with diffused carcinogenic risks, to which most of the planet’s population may be exposed. These carcinogenic risks are represented by *a*) agents that are slightly carcinogenic at any dose; *b*) low or extremely low doses of a carcinogenic agent of any kind; or *c*) mixtures of small doses of carcinogenic agents.

Epidemiologic and experimental studies are fundamental in the identification and quantification of diffused carcinogenic risks, but they must be designed and conducted to be as powerful as possible with adequate methodology. In the case of experimental studies, it is not sufficient to follow the standard protocol used in ordinary experiments. Instead, it is necessary to conduct studies that may be defined as “mega-experiments,” using a vast number of animals (at least 200–1,000 per experimental group) in order to express a marked difference in the variation of effects, and exposing the animals in all phases of development to allow the agent to express its full carcinogenic potential.

It is based on this rationale that the European Ramazzini Foundation performed a mega-experiment on 1,800 rats and demonstrated that, in our experimental conditions, aspartame is a multipotential carcinogenic agent (Soffritti et al. 2005; Soffritti et al. 2006).

The results of our study (Soffritti et al. 2005, 2006) attracted the attention of the scientific community, consumer and industry associations, and the national and international agencies responsible for food safety. Among various comments, the opinion expressed on 5 May 2006 by the European Food Safety Authority (EFSA 2006) and the general interpretation of an epidemiologic study conducted by the National Cancer Institute (NCI 2006) necessitate comment on our part.

In examining the raw data of our study, the EFSA (2006) observed a high incidence of chronic pulmonary inflammation in males and females in both treated groups and in the control group. Based on this observation, it was concluded that “the increased incidence of lymphomas/leukemias reported in treated rats was unrelated to aspartame, given the high background incidence of chronic inflammatory changes in the lungs.” In my opinion, this conclusion is bizarre for the following reasons:

First, the EFSA (2006) overlooked the fact that the study was conducted until the natural death of the rodents. It is well known that infectious pathologies are part of the natural dying process in both rodents and humans.

Second, if the statistically significant increased incidence of lymphomas/leukemias observed was indeed caused by an infected colony, one would expect to observe an increased incidence of lymphomas/leukemias not only in females but also in males. The EFSA (2006) did not comment on this discrepancy in their logic.

Finally, in support of the hypothesis regarding the safety of aspartame, the EFSA (2006) cited the negative results of recent carcinogenicity studies carried out in transgenic mice by the National Toxicology Program (NTP); the ESFA did not mention that, because the NTP studies on genetically altered mice were performed using a new experimental model, the NTP subcommittee unanimously agreed “there is uncertainty whether the study possessed sufficient sensitivity to detect a carcinogenic effect” (NTP 2005).

Interestingly, the same scrutiny applied to our study has not been applied to a recent abstract published by Lim et al. (2006) from the NCI diet questionnaire survey (NCI 2006) in which self-reported aspartame consumption showed no increases in either leukemia/lymphomas or brain cancer. These results have been used by industry, the EFSA, and others to argue that aspartame is not a risk for humans, in spite of our animal study results. Without specific information on each individual’s consumption rate and duration it is difficult to assess the power of the survey, in spite of the large number of participants. The second related issue is whether aspartame is an early-or late-stage carcinogen. If it is an early-stage initiator of cancer, then reporting the lack of effects in older individuals who have not consumed aspartame since early childhood would be expected to show little or no increased cancer (Hoel 1985).

The safety—in particular, the noncarcinogenicity—of today’s most widely diffused artificial sweeteners and their blends is largely based on studies conducted decades ago. I second Karstadt’s nomination of acesulfame K for further study; however, I add that it should be evaluated using a long-term mega-experiment.
